# Systemic Administration of Proteoglycan Protects BALB/c Retired Breeder Mice from Experimental Arthritis

**DOI:** 10.1155/2016/6765134

**Published:** 2016-05-18

**Authors:** Larissa Lumi Watanabe Ishikawa, Priscila Maria Colavite, Thais Fernanda de Campos Fraga-Silva, Luiza Ayumi Nishiyama Mimura, Thais Graziela Donegá França, Sofia Fernanda Gonçalves Zorzella-Pezavento, Fernanda Chiuso-Minicucci, Larissa Doddi Marcolino, Camila Marques, Maura Rosane Valerio Ikoma, Alexandrina Sartori

**Affiliations:** ^1^Department of Microbiology and Immunology, Institute of Biosciences of Botucatu, São Paulo State University (UNESP), 18618-689 Botucatu, SP, Brazil; ^2^Department of Pathology, Botucatu Medical School, 18618-970 Botucatu, SP, Brazil; ^3^Laboratory of Flow Cytometry, Amaral Carvalho Foundation, 17210-120 Jaú, SP, Brazil

## Abstract

This study was undertaken to evaluate the prophylactic potential of proteoglycan (PG) administration in experimental arthritis. Female BALB/c retired breeder mice received two (2xPG50 and 2xPG100 groups) or three (3xPG50 group) intraperitoneal doses of bovine PG (50 *μ*g or 100 *μ*g) every three days. A week later the animals were submitted to arthritis induction by immunization with three i.p. doses of bovine PG associated with dimethyldioctadecylammonium bromide adjuvant at intervals of 21 days. Disease severity was daily assessed after the third dose by score evaluation. The 3xPG50 group showed significant reduction in prevalence and clinical scores. This protective effect was associated with lower production of IFN-*γ* and IL-17 and increased production of IL-5 and IL-10 by spleen cells restimulated* in vitro* with PG. Even though previous PG administration restrained dendritic cells maturation this procedure did not alter the frequency of regulatory Foxp3^+^ T cells. Lower TNF-*α* and IL-6 levels and higher expression of ROR-*γ* and GATA-3 were detected in the paws of protected animals. A delayed-type hypersensitivity reaction confirmed specific tolerance induction. Taken together, these results indicate that previous PG inoculation determines a specific tolerogenic effect that is able to decrease severity of subsequently induced arthritis.

## 1. Introduction

Approximately 1% of people worldwide are affected by rheumatoid arthritis (RA) that is a systemic autoimmune disease [1]. This is a form of arthropathy characterized by a chronic inflammation that leads to progressive cartilage and bone destruction. The etiology of RA is multifactorial, involving both genetic and environmental factors [2]. The immunopathogenetic pathways implicated in RA are not fully elucidated; however, it is known that they involve the recruitment of several cell types from both innate and adaptive immunity [3]. Cytokines are viewed as the main players in arthritic lesions, maintaining chronic inflammation and promoting autoimmunity. TNF-*α* greatly contributes to joint inflammation by stimulating cell proliferation, metalloproteinases and adhesion-molecules expression, secretion of other cytokines, and prostaglandin production by the synovial tissue [4]. IL-6 is another mediator that is found in elevated levels in synovial fluid and serum from RA patients. This cytokine contributes to both local and systemic RA histopathological alterations and clinical manifestations, such as* pannus*, cartilage degradation, fever, fatigue, and weight loss [5].

Some of the autoantigens involved in the pathogenesis of RA include IgG, collagen, proteoglycan, citrullinated proteins, fibrin, fibrinogen, and the shared human leukocyte antigen (HLA) DR epitope [6, 7]. Experimental models induced with some of these antigens, specially collagen and proteoglycan, are being widely employed due to their similarities with the corresponding human disease [8, 9]. We recently demonstrated that bovine PG can also induce typical arthritis when associated with dimethyldioctadecylammonium bromide [10].

It is well known that several autoantigens able to induce autoimmunity have also the potential to induce antigen-specific tolerance depending upon the route, the dose, and the physical form of the antigen [11]. Systemic administration of soluble antigens is a well-accepted approach to induce immunological tolerance [12]. Anergy and clonal deletion of effector cells, induction of regulatory T cells (Tregs), and immune response deviation have been postulated as mechanisms underlying systemic tolerance [11].

To date, RA is not a curable disease and most of the treatments consist in general suppression of the immune response to control synovitis and prevent joint injury worsening [13]. These therapeutic interventions have also side effects including higher susceptibility to infections. Therefore, there is an increasing interest in alternative immunomodulatory strategies, particularly the ones that induce antigen-specific tolerance. Inverse vaccination refers to an antigen-specific immunization protocol aiming at inducing tolerance [14]. These procedures could be, theoretically, the safest measures to avoid or treat autoimmune diseases. In this context, we expected that the systemic administration of proteoglycan (PG) that is a cartilage compound would induce antigen-specific tolerance and therefore restrain arthritis development.

## 2. Material and Methods

### 2.1. Animals

Female BALB/c retired breeder mice aged 8 to 11 months were purchased from CEMIB (Campinas, São Paulo, Brazil). They were maintained in the animal facility of the department of microbiology and immunology under controlled conditions of luminosity (12 h light/12 h dark) and temperature (22 ± 2°C). Mice were allocated in ventilated cages with sterile pine shavings and received sterile food and filtered water* ad libitum*. The manipulation of the animals was in compliance with the local ethics committee (protocol number 257-CEEA).

### 2.2. PG Administration, Arthritis Induction, and Score Evaluation

Mice received two or three intraperitoneal (i.p.) doses of proteoglycan (PG) (50 *μ*g or 100 *μ*g) every three days. One week later, experimental arthritis was induced as described by Ishikawa et al., 2014 [10]. Briefly, mice received three i.p. injections of bovine PG associated with dimethyldioctadecylammonium bromide (DDA) adjuvant at intervals of 21 days. After the third injection, arthritis score was daily evaluated until euthanasia (70 days after the beginning of arthritis induction). Arthritis severity was determined using a standard visual scoring system based on the degree of edema and erytema ranging from 0 to 4 for each paw. The following system was used: 0 = normal; 1 = mild edema in one joint in the paw; 2 = moderate edema and erytema in one or more joints in the paw; 3 = pronounced edema and erytema in all joints in the paw and ankle; 4 = severe edema and erytema of the entire paw and ankle and movement impairment. This classification resulted in a total score that ranged from 0 to 16 for each animal.

### 2.3. Histopathological Analysis

Mice paws were collected after euthanasia and fixed in 10% formalin phosphate buffer for at least 48 hours at room temperature. The samples were demineralized in 10% Titriplex EDTA disodium salt (Merck Millipore, Darmstadt, Germany) for three months. The decalcified tissues were embedded in paraffin and 5 *μ*m sections were mounted on glass slides and stained with hematoxylin and eosin (HE). The images were acquired by a digital camera attached to the optical microscope (Nikon, Kurobanemuko, Otawara, Japan).

### 2.4. Cytokine Production by Spleen Cells

Spleens were collected after euthanasia, ressuspended in RPMI medium containing gentamicin and fetal calf serum (5.0 × 10^6^ cells/mL), and stimulated with PG (50 *μ*g/mL). After 48 hours of incubation at 37°C/5% CO_2_, culture supernatants were collected for TNF-*α*, IL-6, IL-17, IFN-*γ*, IL-5, and IL-10 quantification. These cytokines were assessed by using enzyme linked immunosorbent assay (ELISA), according to manufacturer's instructions (BD Biosciences, San Jose, CA, USA, and RD Systems, Minneapolis, MN, USA). Sensitivity of ELISA kits for these cytokines was 31.25, 19.5, 15.6, 31.25, 7.8, and 15.6 pg/mL, respectively.

### 2.5. Frequency of Dendritic Cells and Regulatory T Cells in the Spleen

Flow cytometry analysis was performed according to manufacturer's instructions (eBiosciences, San Diego, CA, USA). Briefly, for dendritic cells (DCs) immunophenotyping, splenic cells were incubated with FITC-conjugated anti-mouse CD11c (clone N418), APC-conjugated anti-mouse MHCII (clone M5/114.15.2), and PE-conjugated anti-mouse CD80 (clone 16-10A1) antibodies. The cells were washed and ressuspended in flow cytometry buffer containing paraformaldehyde solution. For Tregs, splenic cells were first incubated with FITC-conjugated anti-mouse CD4 (clone GK1.5) and APC-conjugated anti-mouse CD25 (clone PC61.5) antibodies. Intracellular Foxp3 transcription factor was detected using Foxp3 PE Staining Set (eBiosciences, San Diego, CA, USA) according to manufacturer's instructions.

### 2.6. Local mRNA Expression for T Cell Subsets Transcription Factors

mRNA was extracted using TRIzol (Life Technologies) and RNeasy Mini Kit (Qiagen, Valencia, CA, USA). DNase treatment followed manufacturer's instructions and purity of the samples was assessed by 260/280 ratio. Single-strand cDNA synthesis was performed from 100 ng/*μ*L of extracted RNA, using the High Capacity cDNA Reverse Transcription Kit (Applied Biosystems, Carlsbad, CA, USA). Aliquots of cDNA (3 *μ*L) were subjected to real time PCR reaction using TaqMan system. Each reaction contained 15 *μ*L of TaqMan Gene Expression Master mix (Life Technologies, Carlsbad, CA, USA), 0.25 *μ*L of the reference gene, and 1.0 to 1.5 *μ*L of the target genes. The following inventoried primes/probes tested by Life Technologies were used: *β*-actin (Mm00607939_s1), T-bet (Mm00450960_m1), GATA-3 (Mm00484683_m1), ROR-*γ* (Mm01261022_m1), and Foxp3 (Mm00475162_m1). The reactions were performed in ABI 7300 equipment (Applied Biosystems, Carlsbad, CA, USA) using standard parameters. Data were analyzed in SDS Software System 7300 and relative quantification was determined based on fold difference (2^−ΔΔCt^) using Ct value of the target gene normalized to the reference gene and the control (−) group as the calibrator.

### 2.7. Local Cytokine Production

Proteins from paws were extracted by homogenization in RIPA (Radioimmunoprecipitation Assay) buffer with an automatic homogenizer (Ultra Turrax Werke, IKA, Staufen, Germany). Homogenates were centrifuged, the supernatants were filtered through a 22 *μ*m filter, and protein concentration was determined by Pierce method (Thermo Scientific, Waltham, MA, USA). The samples were stored at −80 until cytokines were quantified by Cytometric Bead Array (CBA). Mouse Th1/Th2/Th17 Cytokine Kit (BD Biosciences, San Jose, CA, USA) was used to quantify these cytokines in all samples. Briefly, 25 *μ*L of supernatants was incubated with beads of different sizes conjugated with fluorochromes. The acquisition of the beads was performed in FACS Canto II flow cytometer (BD Biosciences, San Jose, CA, USA) with FACS Diva software and the analysis was done using FCAP 3.0 software (Soft Flow Inc., St. Louis Park, MN, USA).

### 2.8. Delayed-Type Hypersensitivity Reaction (DTH)

Mice were injected with three i.p. doses of 50 *μ*g of PG or chicken ovalbumin (OVA) (Sigma Aldrich, St. Louis, MO, USA) every three days. One week after the last dose, the animals were immunized with a single i.p. dose of PG associated with the DDA adjuvant. DTH was performed one week later by PG inoculation (10 *μ*g) in the hind paw. Paw thickness was measured before and 24 hours after PG inoculation using a caliper (Starrett, Athol, MA, USA).

### 2.9. Statistical Analysis

Results were presented as mean ± standard deviation for parametric variables and the comparison among the groups was performed by ANOVA followed by Tukey's test. For nonparametric variables, the results were presented as median and the comparison among the groups was performed by Kruskal-Wallis followed by Dunn's test. Disease prevalence and severity were compared using Chi-square test. All data were analyzed using SigmaPlot software version 12.0 (Systat Software Inc., San Jose, CA, USA) and *p* < 0.05 was considered significant.

## 3. Results

### 3.1. PG Administration Decreases Arthritis Prevalence and Severity

**Figure 1:**
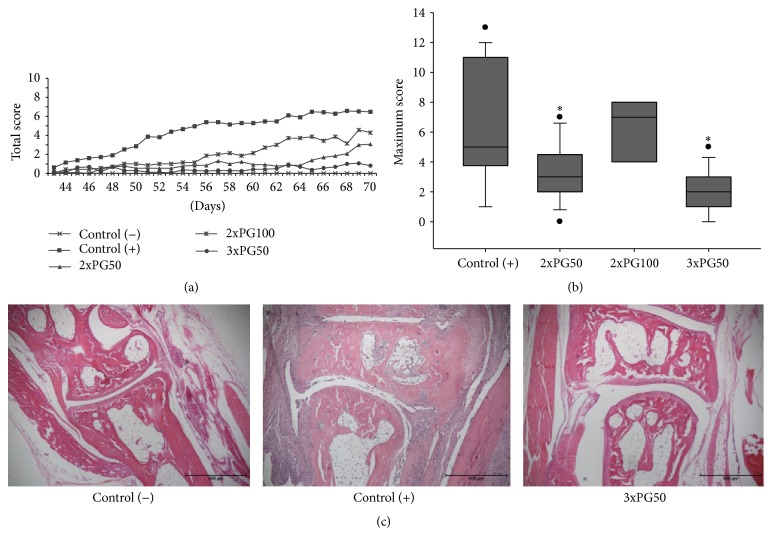
Effect of previous PG inoculation on experimental arthritis development. (a) Kinetics of total score, ranging from 0 to 16 for each animal, assessed by a visual scoring system based on the degree of edema and erytema. (b) Maximum score mean per group. (c) Histopathological analysis assessed 70 days after arthritis induction. Control (−): healthy group not previously injected with PG; control (+): arthritic group not previously injected with PG; 2xPG50, 2xPG100, and 3xPG50: arthritic groups previously injected with two (50 *μ*g), two (100 *μ*g), or three (50 *μ*g) PG doses, respectively. Six to ten animals per group from one representative experiment of two performed. ^*∗*^ *p* < 0.05.

Disease prevalence in 2xPG50 and 2xPG100 groups was similar to those in the control (+) group. However, a significant decrease in prevalence was observed in the group previously injected with 3xPG50 as presented in Table 1. Although clinical disease appeared around days 43 or 44 after arthritis induction in all experimental groups, the maximum clinical scores reached in 2xPG50 and 3xPG50 groups were significantly reduced in comparison to control (+) group. Moreover, these two groups presented a much less severe disease, with a significant smaller number of animals with clinical score above 8. Protection in the 3xPG50 group was also confirmed by histopathological analyses that indicated a clear reduction in inflammation.  Figure 1(c) shows representative micrographs of mice hind paws in score 0 from control (−), score 3 from control (+), and score 1 from 3xPG50 group, respectively. 70 days after arthritis induction, there were a massive inflammation and* pannus* formation only in the control (+) group. The 3xPG50 group presented well preserved joint structures similar to the control (−) group.

### 3.2. PG Administration Reverses the Proportion of Pro- and Anti-Inflammatory Cytokines

**Figure 2:**
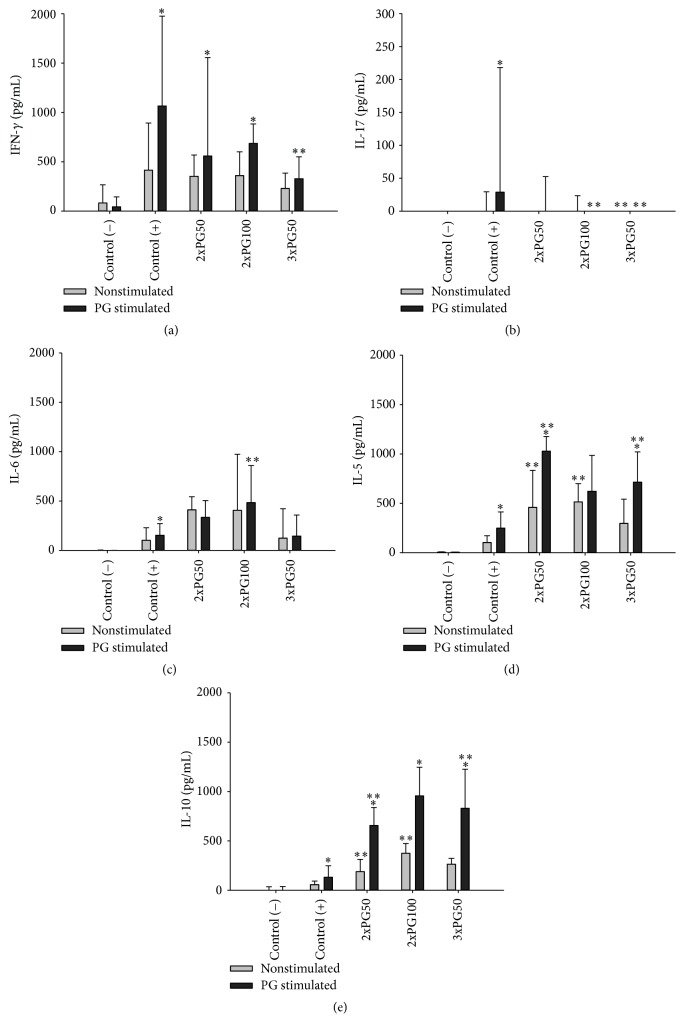
Effect of previous PG inoculation on cytokine production by spleen cells from arthritic mice. (a) IFN-*γ*, (b) IL-17, (c) IL-6, (d) IL-5, and (e) IL-10 production by spleen cells assessed by enzyme linked immunosorbent assay. Control (−): healthy group not previously injected with PG; control (+): arthritic group not previously injected with PG; 2xPG50, 2xPG100, and 3xPG50: arthritic groups previously injected with two (50 *μ*g), two (100 *μ*g), or three (50 *μ*g) PG doses, respectively. Six to ten animals per group from one representative experiment of two performed. ^*∗*^ *p* < 0.05 compared with the unstimulated counterpart and ^*∗∗*^ *p* < 0.05 compared with the control (+) group.

Cytokine profile, which is illustrated in Figure 2, was assessed in both unstimulated and PG stimulated spleen cell cultures. TNF-*α* was not detected in any experimental group (not shown). Spontaneous production of IFN-*γ*, IL-6, IL-5, and IL-10 was observed in all arthritic groups. However, except IL-6, all other cytokines were produced in much higher levels after PG stimulation. Previous administration of PG reduced IFN-*γ* and IL-17 and increased IL-5 and IL-10 production. Reduction in IFN-*γ* and IL-17 levels was more accentuated in the 3xPG50 whereas IL-5 and IL-10 levels were comparable in the three groups inoculated with PG before disease induction.

### 3.3. PG Administration Limited DC Maturation

**Figure 3:**
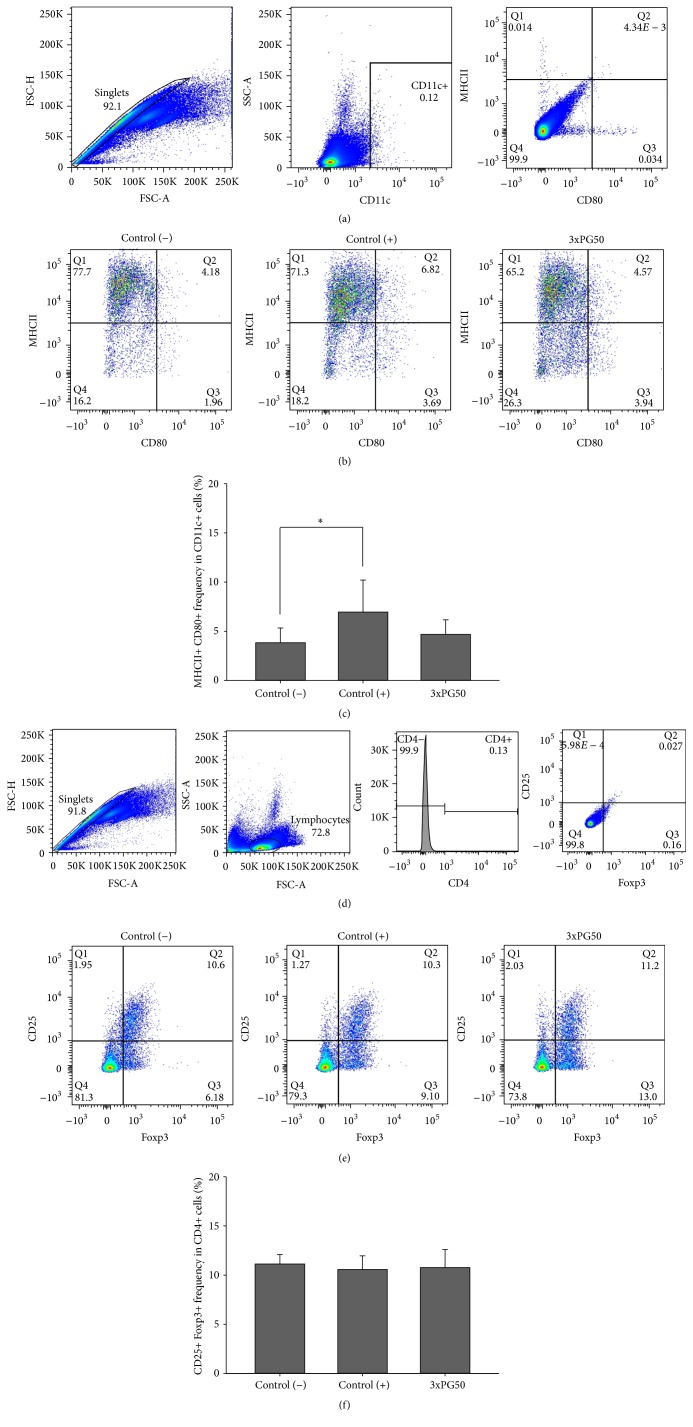
Effect of previous PG inoculation on the frequency of splenic dendritic cells (DCs) and regulatory T cells (Tregs). (a) Gate strategy for DCs in a control (−) sample. (b) Representative flow cytometry analysis for DCs frequency in spleen. (c) MHCII+ CD80+ cell frequency in total CD11+ cells. (d) Gate strategy for Tregs. (e) Representative flow cytometry analysis for Tregs frequency in spleen. (f) CD25+ Foxp3+ cell frequency in total CD4+ cells. Control (−): healthy group not previously injected with PG; control (+): arthritic group not previously injected with PG; 3xPG50: arthritic group previously injected with three doses of PG (50 *μ*g). Six to ten animals per group from one representative experiment of two performed. ^*∗*^ *p* < 0.05.

As DC immaturity has been associated with tolerance induction in rheumatoid arthritis [15], we evaluated the frequency of mature DCs in the spleen of healthy, arthritic, and 3xPG50 experimental groups. The frequency of splenic CD11c+ MHCII+ CD80+ DCs, which is illustrated in Figure 3(c), is significantly higher in the control (+) group whereas the 3xPG50 group presented levels comparable to the control (−) group. Considering that immaturity of DCs is frequently associated with the development of CD4+ CD25+ Foxp3+ T cells [16] and that this is an important mechanism to keep peripheral tolerance, the amount of splenic CD4+ CD25+ Foxp3+ Tregs was also evaluated. However, the frequency of this T cell subset was similar in the three experimental groups (Figure 3(f)).

### 3.4. PG Administration Alters the Proportion of Canonical T Cell Transcription Factors and Drops Down TNF-*α* and IL-6 Levels

**Figure 4:**
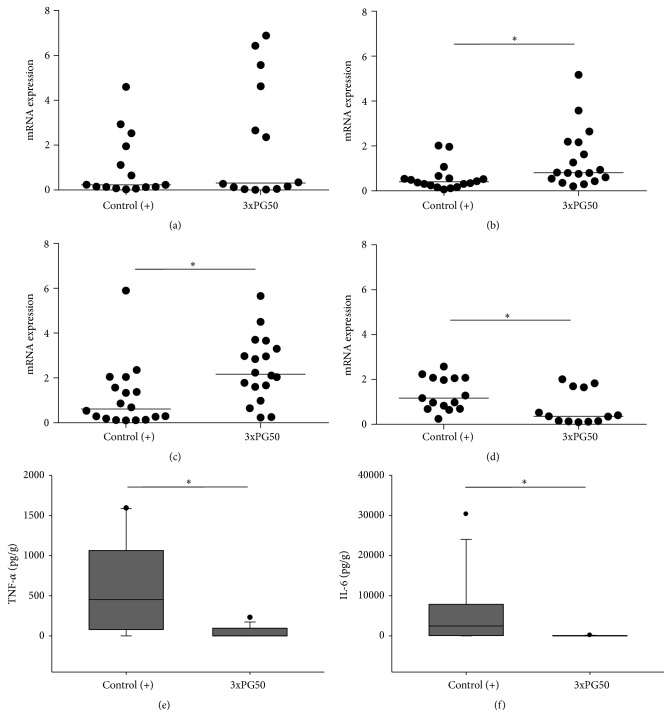
Effect of previous PG inoculation on local T cell transcription factors and cytokine levels. (a) T-bet, (b) ROR-*γ*, (c) GATA-3, and (d) Foxp3 mRNA expression in paw homogenates. Quantification was based on fold difference (2^−ΔΔCt^) between groups, using control (−) group as calibrator. (e) TNF-*α* and (f) IL-6 levels in paw homogenates assessed by Cytometric Bead Array. Control (+): arthritic group not previously injected with PG; 3xPG50: arthritic group previously injected with three doses of PG (50 *μ*g). Hind and forepaws from six to ten animals per group from one representative experiment of two performed. ^*∗*^ *p* < 0.05.

The knowledge of the interplay among T cell subsets has been pivotal to understand arthritis immunopathogenesis and also to suggest new therapeutic modalities [17]. To evaluate if the prophylactic efficacy of PG administration would affect the proportion of these subsets, the local mRNA expression of their respective putative transcription factors was determined. Relative quantification of T-bet, GATA-3, ROR-*γ*, and Foxp3 expression, which are related to Th1, Th2, Th17, and Treg subsets, respectively, was determined. As illustrated in Figure 4, there was a significant increase in GATA-3 and ROR-*γ* expression in animals from the 3xPG50 group compared with the control (+) one. Furthermore, Foxp3 expression was significantly decreased in this previously immunized group when compared with the control (+) group. IL-2, IL-4, IL-10, IL-17, and IFN-*γ* production was not detected in the arthritic joints. However, TNF-*α* and IL-6 levels were detected in all the experimental groups. Previous PG immunization determined a significant reduction in the local (paws) production of these cytokines. These data are also illustrated in Figure 4.

### 3.5. Tolerogenic Effect of PG Administration Is Specific

**Figure 5:**
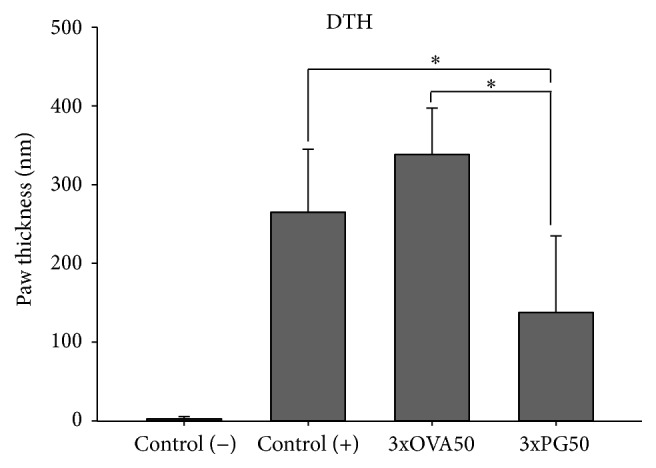
Delayed-type hypersensitivity reaction elicited by PG in mice previously inoculated with PG or OVA. Paw thickness was measured before and 24 hours after PG challenge using a caliper. Control (−): healthy group not previously injected with PG; control (+): arthritic group not previously injected with PG; 3xPG50: arthritic group previously injected with three doses of PG (50 *μ*g). Four to six animals per group from one representative experiment of two performed. ^*∗*^ *p* < 0.05.

A delayed-type hypersensitivity (DTH) reaction was used to confirm that PG was able to induce tolerance and that this tolerance was specific for PG. As expected, there was no increase in the paw thickness of animals from the control (−) group (not immunized with PG+DDA but challenged with PG). However, all other experimental groups presented elevated paw thickness 24 hours after PG challenge in comparison to the control (−) group. Inoculation of OVA (an irrelevant antigen) before immunization with PG+DDA did not trigger a tolerogenic effect. Animals from this group presented paw thickness similar to the control (+) group when challenged with PG. Previous inoculation of the specific antigen (PG) determined a tolerogenic effect demonstrated by a significant decrease in DTH response in comparison to the control (+) group (Figure 5).

## 4. Discussion

Autoimmune diseases such as rheumatoid arthritis, multiple sclerosis, and type 1 diabetes are on the list of the most relevant causes of female death [18]. In this context, much effort has been expended to induce tolerance to autoantigens as an alternative to current and usually global immunosuppressive treatments. Antigen administration by oral route, for example, has been promising by showing a protective effect in experimental diabetes [19] and encephalomyelitis [20]. Intravenous administration of soluble antigen in the absence of adjuvants is another well documented procedure to induce tolerance [21, 22]. The present study was undertaken to evaluate if proteoglycan (PG) injected by intraperitoneal (i.p.) route could induce a tolerogenic state and consequently reduce arthritis development. We initially demonstrated that i.p. PG administration, before arthritis induction by this same antigen, was able to decrease disease prevalence, clinical score, and also the histopathological features. The most effective protocol was the inoculation of three doses of 50 *μ*g of PG. Two doses of 50 *μ*g of PG triggered a similar but less accentuated protective effect. The prophylactic effect observed in this work is consistent with the literature. Already in 1997, Liblau et al. [12] suggested that systemic inoculation of soluble proteins, synthetic peptides, or peptide analogues could become an acceptable approach to prevent or to treat human autoimmune diseases as multiple sclerosis and type 1 diabetes. The prophylactic effect of a cartilage specific antigen was described, for the first time, by Gumanovskaya et al., 1999 [23]. At first sight, it could appear too complex to translate our findings to human arthritis because, differently from multiple sclerosis and type 1 diabetes, whose involved autoantigens are better established, arthritis targeted autoantigens are still not so well characterized. However, it was recently reported that oral administration of shark type II collagen suppressed arthritis induced in rats by inoculation of Complete Freund's Adjuvant [24]. It is possible, therefore, that a specific joint antigen, as is the case of PG, can trigger a similar protective effect in human arthritis when administered by a systemic route. This possibility still needs a profound preclinical investigation.

To elucidate the possible protective mechanism triggered by PG, we initially analyzed the production of some pro- and anti-inflammatory cytokines. All experimental groups produced detectable levels of IFN-*γ* and IL-17 but not TNF-*α*. These findings are in accordance with several studies that reveal the proarthritogenic effect of IFN-*γ* and IL-17 [25]. Previous PG administration prevented the development of a more severe disease and concomitantly reduced the production of IFN-*γ* and IL-17. Analysis of cytokine production by spleen cells stimulated with PG also suggests that the protective effect is mediated by an increased production of IL-5 and IL-10 anti-inflammatory cytokines. As IL-5 is described as a typical Th2 cytokine [26, 27], it is possible that i.p. administration of PG has polarized the immune response towards Th2. The higher expression of GATA-3 in the paws from previously PG injected mice reinforces this possibility. Moreover, IL-10 has been described by some authors as being comparable to IL-4 and IL-5 concerning the Th2 pattern [28]. IL-10 is endowed with a strong anti-inflammatory potential and its contribution to tolerance induction to both self-antigens and foreign antigens has also been described [29]. A similar anti-inflammatory role has been attributed to these cytokines in experimental diabetes [30] and encephalomyelitis [31]. The higher percentage of mature DCs in the positive control group shows that the arthritogenic process is associated with an increased maturation of DCs. Otherwise, the smaller amount of mature DCs in PG inoculated mice suggests that PG effect over DCs could be involved in this protective effect. Demonstration that skewing DCs differentiation towards a tolerogenic state is efficient as an RA treatment supports this possibility [15]. Unexpectedly, however, there was no increase in the percentage of Tregs. A possible interpretation of these findings is that PG administration by i.p. route is determining immunological tolerance independently of Tregs. Intraperitoneal delivered substances are primarily absorbed by mesenteric vessels which drain into the portal vein and pass to the liver. It is well known that this organ also uses other pathways to establish a state of immunological tolerance [32]. Satpute et al. 2009 [33] that employed the i.p. route did not observe alterations in Tregs frequency and function in rats with adjuvant arthritis after treatment with the 65 kDa heat shock protein. They also demonstrated that this tolerogenic effect was not mediated by indoleamine 2,3-dioxygenase expressing DCs.

Otherwise, the increased expression of GATA-3 mRNA levels suggests higher accumulation of Th2 polarized cells in the joints of PG protected mice. This is consistent with the literature since Th2 polarization has been associated with tolerance induction by systemic immunization [11]. The increased expression of ROR-*γ* and the decreased expression of Foxp3 in the tolerized experimental group were unexpected since Th17 and Treg subsets are usually associated with antagonistic roles in the course of autoimmune inflammatory pathologies [34]. The plasticity phenomenon that characterizes T cell subsets could account for this finding. The extreme complexity of Tregs plasticity is being acknowledged [35] and includes an unpredictable correlation between Foxp3 expression and suppressive ability and also the potential of this subset to acquire the features of effector T cells [36]. Accumulating evidences also indicate that Th17 cells are particularly prone to present functional plasticity. Thus, additional subsets as Th17/Th1, Th17/Th2, and Th17/Treg have been recently described [37]. The concomitant elevated expression of GATA-3 and ROR-*γ* could therefore represent the local presence of a Th17/Th2 subset. Alternatively, the elevated amount of Th cells expressing ROR-*γ* (Th17) could be linked to Tregs plasticity. In this scenario, Tregs that migrated early to the lesions could later express the Th17 transcription factor. This possibility is partially supported by a recent report by Wang et al. 2015 [38]. These authors demonstrated that Tregs from RA patients presented increased plasticity toward Th17. They also observed that IL-17 producing T cells retained suppressive function and were associated with milder inflammatory conditions.

A possible correlation between T cell subsets and their signature cytokines was not found in mice paws, but the prophylactic PG effect clearly downmodulated the local production of TNF-*α* and IL-6. The relevance of these findings resides in the pivotal role of these two mediators on joint destruction [39]. This knowledge upgraded disease treatment with the adoption of various strategies based, initially, on TNF-*α* inhibition [40]. More recently, mainly because some RA patients are nonresponsive to anti-TNF therapy, agents targeting IL-6 are viewed as promising biologicals in RA treatment [41].

To determine if the prophylactic activity elicited by PG administration was specific, we compared the effects of previous injection of PG and OVA on the intensity of a DTH reaction. Previous administration of three doses of the specific antigen (PG) determined a tolerogenic effect characterized by a significant decrease in the DTH reaction in comparison to the control (+) group. This effect was not observed in animals previously inoculated with the nonrelated cartilage antigen (OVA), indicating, therefore, that the tolerogenic effect was specific for PG. The possible prophylactic use of bovine PG to prevent human arthritis, based on the presence of shared epitopes between human and bovine PG [42], certainly needs further investigation.

Further investigation is also necessary to disclose if this procedure can be translated to RA patients who already present with disease symptoms. Considering that these patients have a compromised self-tolerance, RA putative target autoantigens as PG, collagen, or citrullinated molecules could be associated with tolerogenic adjuvants or with the standard RA therapies to optimize disease treatment. The possible therapeutic use of PG alone or combined with vitamin D3 is being investigated by our research group. We also recently demonstrated that treatment with myelin peptide combined with vitamin D3 controlled experimental encephalomyelitis development in both therapeutic [43] and prophylactic [44] approaches. Other reports reinforce the feasibility of tolerogenic approaches in RA therapy [45]. Combined therapies would also allow decreasing immunosuppressive drugs dosage and, therefore, avoiding a generalized immunosuppression [46]. In addition, these antigen-specific strategies could be efficacious during the preclinical disease phase when immunological abnormalities are already happening without clinical manifestation.

## 5. Conclusion

Together, these results indicate that previous PG inoculation determines a specific tolerogenic effect strong enough to reduce the severity of subsequently induced arthritis.

## Figures and Tables

**Table 1:** Arthritis prevalence and severity in mice previously inoculated with proteoglycan. Arthritis score was daily evaluated and disease severity was assessed by a visual scoring system with total score ranging from 0 to 16 for each animal. Score above eight indicates severe arthritis. These parameters were compiled 70 days after arthritis induction. Control (+): arthritic group not previously injected with PG; 2xPG50, 2xPG100, and 3xPG50: arthritic groups previously injected with two (50 *μ*g), two (100 *μ*g), or three (50 *μ*g) PG doses, respectively. Data from two independent experiments were combined.

	Arthritis prevalence	*p* value	Disease onset (days)	Animals with score > 8	*p* value
Control (+)	22/22 (100%)	0.042	43	9/22 (41%)	<0.001
2xPG50	12/13 (92%)		44	0/13 (0%)	
2xPG100	7/7 (100%)		44	0/7 (0%)	
3xPG50	12/16 (75%)		43	0/16 (0%)	
